# Serum miRNA and Metabolomic Signatures of Residential Radon Exposure in Chiang Mai, Thailand

**DOI:** 10.3390/toxics13121021

**Published:** 2025-11-26

**Authors:** Moe Thi Thi Han, Tarika Thumvijit, Chutima Kranrod, Shinji Tokonami, Kanyamas Choocheep, Warunee Kumsaiyai, Yupanun Wuttiin, Khanittha Punturee, Sakorn Pornprasert, Sawitree Chiampanichayakul, Ratchada Cressey

**Affiliations:** 1 Division of Clinical Chemistry, Department of Medical Technology, Faculty of Associated Medical Sciences, Chiang Mai University, Chiangmai 50200, Thailand; moethithihan@gmail.com (M.T.T.H.); kanyamas.c@cmu.ac.th (K.C.); warunee.kumsaiyai@cmu.ac.th (W.K.); khanittha.taneyhill@cmu.ac.th (K.P.); 2 Department of Radiologic Technology, Faculty of Associated Medical Sciences, Chiang Mai University, Chiangmai 50200, Thailand; tarika.thumvijit@cmu.ac.th; 3 Institute of Radiation Emergency Medicine, Hirosaki University, Hirosaki-shi 036-8560, Japan; kranrodc@hirosaki-u.ac.jp (C.K.); tokonami@hirosaki-u.ac.jp (S.T.); 4 Cancer Research Unit, Department of Medical Technology, Faculty of Associated Medical Sciences, Chiang Mai University, Chiangmai 50200, Thailand; yupanun.wuttiin@cmu.ac.th (Y.W.); sawitree.chiampa@cmu.ac.th (S.C.); 5 Division of Transfusion Science, Department of Medical Technology, Faculty of Associated Medical Sciences, Chiang Mai University, Chiangmai 50200, Thailand; 6 Division of Microscopy, Department of Medical Technology, Faculty of Associated Medical Sciences, Chiang Mai University, Chiangmai 50200, Thailand; sakorn.pornprasert@cmu.ac.th

**Keywords:** radon, circulating miRNA, serum metabolomics, health, Chiang Mai, miRNA expression profile

## Abstract

Residential radon is a leading environmental cause of lung cancer, but circulating biomarkers linking home exposure to pathogenic biology are not well defined. We conducted an exposure-contrast study in Hang Dong District, Chiang Mai, measuring indoor radon in 48 homes and enrolling adults from <50 Bq/m^3^ (low) and ≥100 Bq/m^3^ (high) households for serum profiling. Mean indoor radon was 61.8 ± 18.4 Bq/m^3^ (range 34–126), with 6.2% of homes ≥100 Bq/m^3^. Small RNA sequencing identified 55 differentially expressed miRNAs (12 up, 43 down) in high-radon serum. Notably, miR-200b-3p, miR-200c-3p, and miR-194-5p were increased, while miR-3913-5p, miR-584-5p, miR-30a-3p, miR-22-3p, and miR-125a-5p were decreased. Target enrichment (KEGG/GO) implicated PI3K–Akt and MAPK hubs with Ras/Wnt/VEGF alongside focal adhesion/ECM–receptor/actin–cytoskeleton and immune-regulatory modules. Untargeted LC–MS metabolomics showed exposure-aligned shifts: higher PUFAs and oxylipins (e.g., AA, EPA; 9-HEPE, 8-HETE, 5,12-DiHETE), elevated acyl-carnitines (β-oxidation), and increased inosine/hypoxanthine, consistent with lipid/steroid remodeling, mitochondrial fuel reprogramming, oxidative stress, and nucleotide turnover. Integrated interpretation supports DDR/ATM → PI3K/Akt–MAPK activation with EMT/adhesion remodeling, angiogenic signaling, and immune modulation—linking residential radon to lung cancer mechanisms. Given the small sample size (*n* = 10), these findings should be interpreted as preliminary and hypothesis-generating, warranting validation in larger cohorts. Nevertheless, findings support household testing, remediation at ≥100 Bq/m^3^, and integrated exposure studies considering PM2.5 co-exposures.

## 1. Introduction

Lung cancer in never-smokers is rising. It now accounts for a growing share of cases and deaths worldwide, especially among women and in Asian populations [1]. Among environmental determinants, residential radon is a well-established cause of lung cancer independent of smoking, and the World Health Organization estimates that radon contributes to approximately 3–14% of lung cancer cases worldwide. Applied to the 2.48 million incident cases reported in 2022, this corresponds to ~74,000–347,000 radon-attributable cases [2]. Recent Global Burden of Disease data also estimate ~82,000 lung cancer deaths in 2021 linked to residential radon, underscoring its global health significance [3].

Chiang Mai, Thailand, offers a relevant setting for radon research due to its geological characteristics and documented elevated indoor radon levels. Reported concentrations range from 29 to 101 Bq/m^3^ in Pa Miang (Doi Saket) and 17–118 Bq/m^3^ in Doi Lo, with thoron levels between 12 and 72 Bq/m^3^, corresponding to ~0.5–5 mSv/year of total progeny dose. Across Upper Northern Thailand, indoor radon levels span 11–405 Bq/m^3^, and approximately 26–28% of lung cancer deaths are attributed to indoor radon exposure [4]. Spatial analyses (2008–2017) further show increased lung cancer mortality risk in western Chiang Mai districts, including Hang Dong, Doi Lo, and San Pa Tong, supporting the region’s suitability for radon-exposure biomarker studies [5].

MicroRNAs (miRNAs) are short non-coding RNAs that regulate gene expression and circulate in stable forms in the blood [6]. They are protected from degradation by encapsulation in extracellular vesicles or association with proteins, allowing reliable measurement from small serum volumes [7]. Because miRNA profiles shift in response to cellular stress, inflammation, and DNA damage, circulating miRNAs serve as minimally invasive indicators of biological responses to environmental exposures [8]. They can be quantified using small RNA sequencing or RT-qPCR [9], and their validated mRNA targets enable direct pathway interpretation of biological pathways.

Serum metabolomics characterizes circulating small molecules that reflect genetic, protein, metabolic, and microbiome activity. It provides a systems-level view of physiological changes and is sensitive to the oxidative stress, inflammation, and metabolic dysregulation associated with environmental exposures [10,11]. High-resolution LC-MS enables scalable detection of coordinated metabolic shifts across lipid, amino acid, and energy pathways [12], supporting exposure assessment and biomarker discovery in population studies [13]. Integrating miRNA and metabolomic data can improve mechanistic inference by determining whether miRNA-regulated pathways align with observed metabolic changes. Emerging exposome research underscores the importance of assessing the cumulative biological impact of multiple environmental factors across the life course [14]. This approach aligns with exposome-focused research priorities, which emphasize the cumulative impact of environmental exposures over the life course. Multi-omics profiling can therefore identify coherent biological signatures of radon exposure and support early-stage biomarker development. Based on this rationale, the present study characterized circulating molecular signatures associated with residential radon exposure by profiling serum miRNAs and metabolites in adults from low- and high-radon households. The objective was to identify differentially expressed miRNAs, perturbed metabolic pathways, and cross-omics concordance between miRNA targets and metabolic alterations.

## 2. Materials and Methods

### 2.1. Study Area and Site Selection

This exposure-contrast study was conducted in two communities in Hang Dong District, Chiang Mai: San Phak Wan (urban) and Nong Kaeo (rural). These sites were selected based on their differences in urbanicity and geological features, while sharing similar climate and cultural characteristics. Hang Dong has previously been identified as a localized high-risk area for lung cancer mortality in Upper Northern Thailand, with nearby districts such as Doi Lo and San Pa Tong also showing elevated risk [5]. This background supports its suitability for environmental exposure and biomarker research.

Radon was monitored at two sites (H1, H2) in both indoor and outdoor environments. Short-term atmospheric radon concentrations were measured using a pulse-ionization chamber (AlphaGUARD PQ2000PRO, Genitron Instruments, Frankfurt, Germany), which recorded hourly values over a two-week period at each site. The detector (range 2–2 × 10^6^ Bq/m^3^) was mounted approximately 1 m above ground level. To assess long-term residential exposure, RADUET passive alpha-track monitors (Radosys, Budapest, Hungary) were installed in 26 households in San Phak Wan and 25 households in Nong Kaeo. RADUET uses two diffusion chambers with different air exchange rates to reduce thoron interference and improve radon estimation accuracy. Units were placed in living rooms at ~0.9–1.2 m above the floor and at least 0.5 m from walls, windows, and heat or humidity sources, following manufacturer guidelines. The monitors were deployed for approximately four months (February–June 2023) to derive the annual average radon concentrations, consistent with WHO European air quality guidance, and were subsequently sealed and analyzed at the Institute of Radiation Emergency Medicine, Hirosaki University (Aomori, Japan). For biomarker analysis, households were classified a priori as low exposure (<50 Bq/m^3^) or high exposure (≥100 Bq/m^3^). Homes with intermediate values (50–99 Bq/m^3^) were not included to maintain a clear exposure contrast.

### 2.2. Participant Eligibility and Recruitment

Adults (≥18 years) who had lived in their current home for ≥12 months first consented to in-home passive radon monitoring. After results became available, households were contacted only if the time-integrated radon level was <50 Bq/m^3^ (low exposure) or ≥100 Bq/m^3^ (high exposure); homes with 50–99 Bq/m^3^ were not eligible. Participants also needed to be available during the sampling window. Homes undergoing major renovation, using atypical ventilation during measurement, or residents with known occupational radon exposure were excluded. All participants provided written informed consent. The study was approved by the AMS-CMU IRB (protocol AMSEC-66EX-004).

### 2.3. Blood Collection and Processing

After a minimum 8–12 h fast, about 6–10 mL of venous blood was collected from 07:00 to 10:00 into EDTA tubes, kept at 4 °C, and transported to AMS-CMU within 2 h. Plasma was separated by centrifugation (1500–2000× *g*, 10 min, 4 °C), aliquoted into RNase-free tubes (≥200 µL per aliquot), and stored at −80 °C until analysis. One aliquot per participant was used for small RNA library preparation, and a separate aliquot was reserved for label-free proteomics to avoid freeze–thaw cycles.

### 2.4. Small RNA Sequencing

Small RNA sequencing was conducted for 12 participants (low radon, *n* = 6; high radon, *n* = 6). During post-enrollment verification, two participants were identified as active smokers and were excluded a priori from all omics analyses to minimize smoking-related confounding. The final analytic cohort was 10 participants (low radon, *n* = 4; high radon, *n* = 6). Serum miRNA was extracted using the NucleoSpin^®^ miRNA Plasma/Serum kit (Macherey-Nagel, Düren, Germany) per manufacturer’s instructions. Approximately 10 ng of total small RNA per sample was submitted to NovogeneAIT Genomics (Singapore) for library preparation and Illumina-compatible sequencing, generating single-end reads at a depth sufficient to detect low-abundance miRNAs. Primary processing used Trimmomatic v0.30 for adapter trimming and quality filtering (reads with average quality <20 and length <18 nt discarded). Clean reads were analyzed with miRDeep2 to align to the human reference genome, annotate known miRNAs (miRBase), and predict novel candidates from hairpin structures.

Expression values (raw counts) were imported into DESeq2 (Bioconductor version 1.34.0.) for normalization and differential expression analysis between low- and high-radon groups. Wald test *p*-values were adjusted for multiple testing using the Benjamini–Hochberg false discovery rate (FDR) procedure to obtain adjusted *p*-values (padj). For exploratory analyses, miRNAs with an absolute log2 fold change (high vs. low radon) ≥ 1 and nominal *p* < 0.05 were considered radon-responsive and were used for clustering and GO/KEGG enrichment.

### 2.5. RT-qPCR Validation of Selected miRNAs

To technically validate key miRNA signals identified by small RNA sequencing, RT-qPCR was performed on serum from the same 10 non-smoking participants included in the sequencing analysis. Total small RNA was extracted as described above. For quantification of miRNA expression levels, cDNA synthesis was carried out using a polyadenylation-based reverse-transcription method, followed by qPCR using iTaq™ Universal SYBR^®^ Green Supermix on a CFX96 Touch™ Real-Time PCR Detection System (Bio-Rad, Hercules, CA, USA). Specific primer pairs were used for miR-200b-3p, Novel-miRNA-203, miR-194-5p, and miR-584-5p. miR-484 was used as the endogenous control because of its stable expression across samples. The relative expression of each miRNA was determined using the 2^−ΔCt^ method, where ΔCt represents the difference between the average cycle threshold (Ct) of the target miRNA and the average Ct of miR-484 in the same sample. All reactions were run in duplicate, and mean Ct values were used for analysis. RT-qPCR results are presented in Supplementary Figure S1.

### 2.6. Untargeted Serum Metabolomics Analysis

Serum metabolites were extracted by mixing 100 µL serum with 300 µL methanol, vortexing for 30 s, and standing at room temperature for 20 min. Samples were centrifuged at 16,000× *g* for 15 min at 4 °C. Supernatants were collected and evaporated at 40 °C for ~25 min to dryness. Dried extracts were reconstituted in 100 µL water containing 25 ng/mL of a four-sulfa internal-standard mix. From each sample, 30 µL was pooled to prepare a QC and a seven-point dilution QC series (0%, 1%, 10%, 20%, 50%, 80%, 100%). After centrifugation at 14,000 rpm for 10 min, supernatants were transferred to LC–MS vials.

Chromatography was performed using a Poroshell 120 EC-C18, 2.1 × 100 mm, 2.7 µm column at 50 °C with a 10 µL injection. Mobile phase A was 0.1% formic acid in water, and mobile phase B was 0.1% formic acid in acetonitrile at 0.4 mL/min. The gradient started at 98% A/2% B (0 min), was increased to 2% A/98% B (18 min), held for 23 min, returned to 98% A/2% B (23.5 min), and re-equilibrated for 28 min. Data were acquired in positive and negative ion modes on an Agilent 6495 triple quadrupole LC–MS (Baiya Phytopharm, Chulalongkorn University (Bangkok, Thailand)). Source parameters were as follows: drying gas 325 °C, 13 L/h; sheath gas 275 °C, 12 L/h; nebulizer 45 psi; capillary 4000 V; isotope width 1.3 *m*/*z*. MS1 range was 40–1700 m/z and MS2 range was 25–1000 *m*/*z* with collision energies of 20 eV (positive) and 10 eV (negative). Acquisition rate was 3.35 spectra/s, with up to 10 precursors per cycle, a precursor threshold of 5000 counts, and a retention time threshold of 0.001%. Reference masses were 121.0509 and 922.0098 *m*/*z* (positive) and 112.9856 and 1033.9881 *m*/*z* (negative).

Raw files were processed in MS-DIAL v5.3. Alignment was performed against the pooled QC, and peak intensities were normalized to sulfadimethoxine (*m*/*z* 311.0810 in positive mode; 309.0047 in negative). The following masses were excluded: 121.0509 and 922.0098 *m*/*z* (positive) and 112.9856, 119.0363, 966.0007, and 1033.9881 *m*/*z* (negative). Adduct searches included [M+H]+ (positive) and [M−H]− (negative). Annotation was performed using the MS-DIAL ESI (±) MS/MS database with authentic standards. Metabolite identification was performed using accurate mass, retention time, isotope pattern, and MS/MS spectral similarity, with MS1 (40–1700 *m*/*z*) and MS2 (25–1000 *m*/*z*) matching at mode-specific collision energies (positive: 20 eV; negative: 10 eV). For peak assignment, MS1 mass accuracy tolerance was set to ±0.01 Da, and MS2 fragment-ion tolerance was set to ±0.025 Da, following the default high-resolution settings in MS-DIAL v5.3. Isotope matching was performed using a width tolerance of 1.3 m/z, and retention time alignment was based on the pooled QC injection. Features were accepted only if Pearson r ≥ 0.8 across dilution QCs, QC %CV < 30%, and MS-DIAL identification score ≥ 0.8. Based on these criteria and the use of authenticated MS/MS spectra, annotations correspond to MSI Level 2 (putatively annotated compounds) and MSI Level 1, where matching RT and MS/MS spectra were available. After filtering, 4629 features remained in positive mode and 2102 features in negative mode.

Peak tables were exported to MetaboAnalyst 6.0 for statistical analysis. Data were treated as unpaired and log_10_-transformed; no additional filtering was applied. For univariate statistical analysis, two-group comparisons were performed, and raw (unadjusted) *p*-values were reported. Because of the small sample size and exploratory design, *p*-values were not corrected for multiple testing and are interpreted in a hypothesis-generating context. Metabolite features were considered differentially abundant when they met both *p* < 0.05 and |log2(fold change)| ≥ 1.

## 3. Results

### 3.1. Household Radon Concentrations and Selection of Study Participants

**Figure 1:**
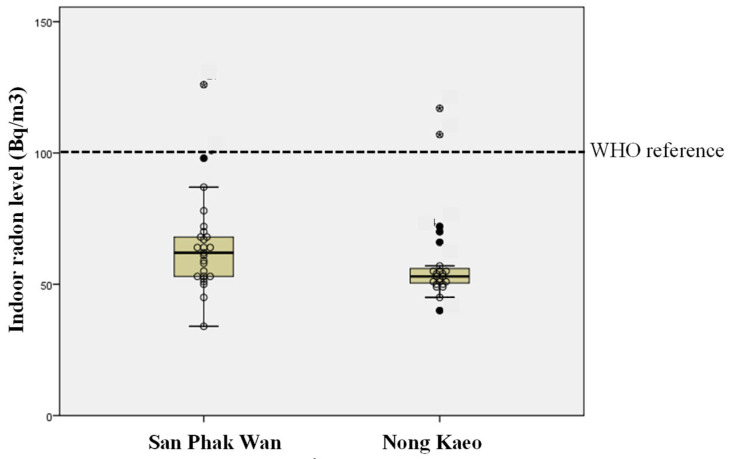
Indoor radon concentrations in two Hang Dong communities. Box-and-whisker plots show household measurements for San Phak Wan (*n* = 25) and Nong Kaeo (*n* = 23). The dashed line denotes the WHO indoor radon reference threshold (100 Bq/m^3^). Each dot represents the indoor radon level measured in an individual household.

Indoor radon was measured in 48 households across the two study areas (Table 1; Figure 1). In San Phak Wan (*n* = 25), the mean concentration was 64.5 ± 18.4 Bq/m^3^ (range 34–125 Bq/m^3^). In Nong Kaeo (*n* = 23), the mean was 60.0 ± 18.3 Bq/m^3^ (range 40–117 Bq/m^3^). Overall, the combined mean across both areas was 61.8 ± 18.4 Bq/m^3^ (range 34–126 Bq/m^3^). As shown in Figure 1, both communities exhibited similar distributions centered near 60 Bq/m^3^, with a few higher values.

Using the WHO reference level of 100 Bq/m^3^, 45 of 48 households (93.8%) were below this threshold, and 3 households (6.2%) were at or above it (one in San Phak Wan and two in Nong Kaeo). From these households, six volunteers from high-radon homes (≥100 Bq/m^3^) and six volunteers from homes below 50 Bq/m^3^ were selected for downstream molecular analyses.

### 3.2. Differential Expression of miRNAs Between Low and High Radon Exposure

The low- and high-radon groups showed broadly comparable demographic characteristics. All ten participants were non-smokers, and all blood samples were collected on the same date (14 October 2023), minimizing temporal variation. The low-radon group (*n* = 4) had a mean age of 51.0 ± 3.46 years and included two males and two females, whereas the high-radon group (*n* = 6) had a mean age of 49.7 ± 6.71 years and included two males and four females. Mean BMI was 22.1 ± 0.56 kg/m^2^ in the low-radon group (range 21.6–22.6) and 24.0 ± 3.00 kg/m^2^ in the high-radon group (range 22.0–29.7). One participant in the high-radon group had a BMI of 29.7 kg/m^2^ (obesity range). Occupations varied across both groups (municipal employees, farmers, businessmen, merchants) with no apparent clustering by radon exposure category.

**Figure 2:**
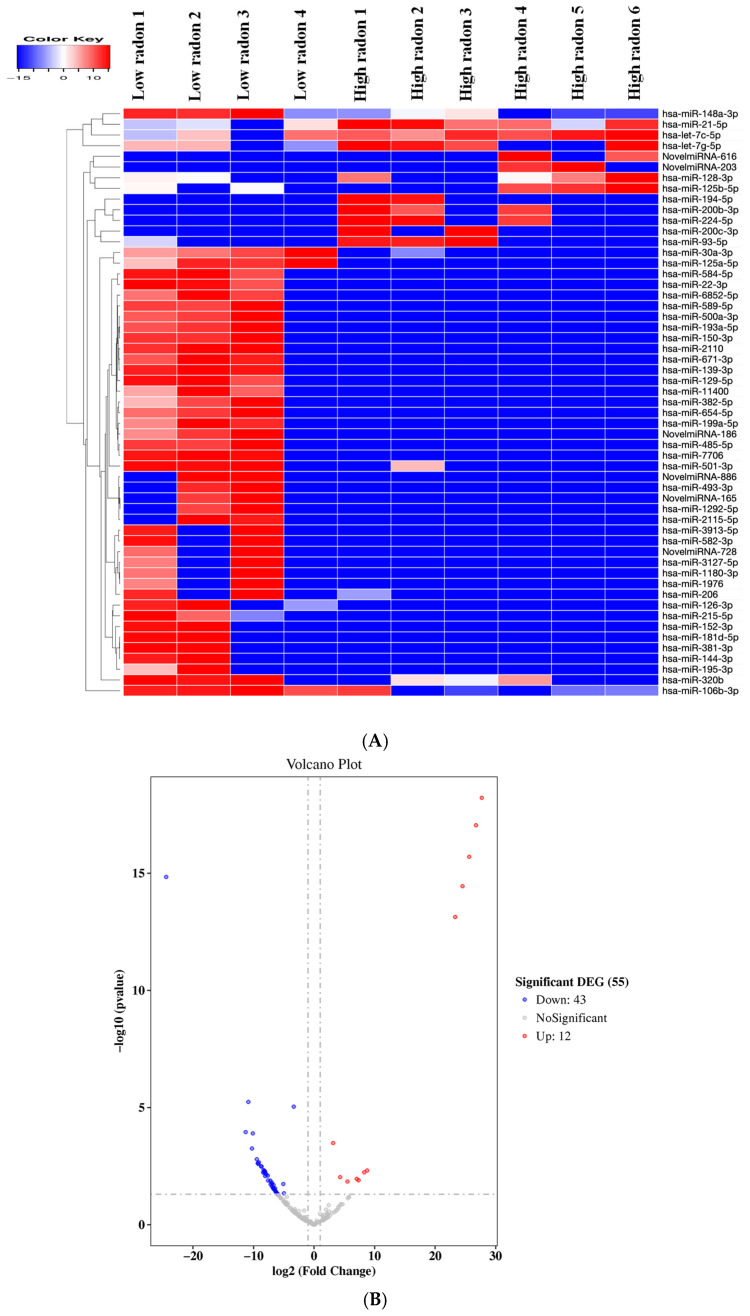
Differentially expressed miRNAs between low- and high-radon groups. (**A**) Heat map showing normalized expression of differentially expressed miRNAs, with unsupervised hierarchical clustering separating participants by exposure status. (**B**) Volcano plot depicting differential expression (high vs. low radon); the horizontal dashed line indicates significance (*p* < 0.05) and vertical dashed lines mark effect-size cutoffs (|log2FC| ≥ 1). Red and blue points represent significantly upregulated (*n* = 12) and downregulated (*n* = 43) miRNAs, respectively, for a total of 55 altered miRNAs.

Circulating miRNAs were profiled from the serum using small RNA sequencing. A hierarchical clustering heatmap showed a clear separation between participants from low- and high-radon households (Figure 2A). Samples from the same exposure group clustered closely together, indicating strong within-group consistency. A subset of miRNAs was more highly expressed in the low-radon group, whereas these same miRNAs were suppressed in the high-radon group. Conversely, several miRNAs were up-regulated in the high-radon group while remaining low in the low-radon group, demonstrating distinct exposure-related expression patterns.

Differential expression analysis performed using DESeq2 identified 55 radon-responsive miRNAs, defined by an absolute log2 fold change ≥ 1 and nominal *p* < 0.05 (Figure 2B). Several miRNAs showed large effect sizes, and the predicted novel miRNAs from miRDeep2 followed the same exposure-aligned trends observed in the heatmap. A subset of these miRNAs also met an FDR-adjusted significance threshold (Benjamini–Hochberg padj < 0.05) and is listed in Table 2.

Using a more stringent cut-off (|log2FC| ≥ 10 with FDR control), hsa-miR-200b-3p, hsa-miR-200c-3p, and hsa-miR-194-5p emerged as the most strongly up-regulated miRNAs in high-radon serum. The most strongly down-regulated were hsa-miR-3913-5p, hsa-miR-584-5p, hsa-miR-30a-3p, hsa-miR-22-3p, and hsa-miR-125a-5p. Two predicted novel miRNAs (NovelmiRNA-616 and NovelmiRNA-203) also met this threshold. These high-magnitude changes support the exposure-related contrast observed in the heatmap and volcano plot (Figure 2A,B) and were prioritized for follow-up.

Together, these results suggest that chronic household radon exposure is associated with coordinated shifts in circulating small RNAs, with repression being the predominant response. Given the modest sample size and the exclusion of smokers to minimize confounding, these findings should be interpreted as discovery stage observations and will be prioritized for targeted validation and functional follow-up. Although one high-radon participant had a BMI in the obesity range, their values fell within the overall distribution of the group, and excluding this individual did not alter the direction or magnitude of the radon-associated miRNA signatures.

### 3.3. RT-qPCR Validation of Radon-Responsive miRNAs

RT-qPCR was used to validate four representative miRNAs identified from the sequencing analysis (miR-200b-3p, Novel-miRNA-203, miR-194-5p, and miR-584-5p). As shown in Supplementary Figure S1, the direction of change measured by RT-qPCR was consistent with the small RNA-seq results: miR-200b-3p, Novel-miRNA-203, and miR-194-5p tended to show higher relative expression in participants from high-radon households, whereas miR-584-5p tended to be lower in the high-radon group. Although these differences did not reach statistical significance because of the small sample size (*n* = 4 and 6), the concordant trends support technical validation of the key radon-responsive miRNA candidates.

### 3.4. Gene Ontology (GO) Enrichment Analysis

**Figure 3:**
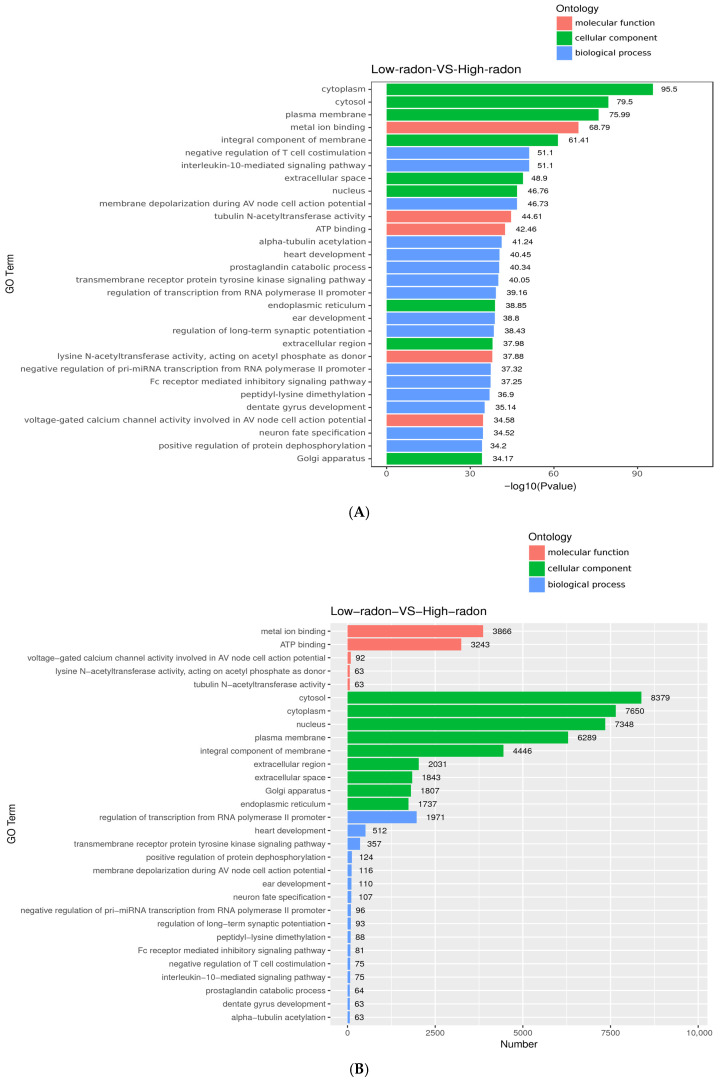
Gene Ontology (GO) enrichment analysis of predicted targets of radon-responsive miRNAs. (**A**) Bar plot of enriched GO terms ranked by −log10 *p*-value across biological process, cellular component, and molecular function categories. (**B**) Corresponding hit counts for the same terms, illustrating broad enrichment of membrane/secretory and ion-handling gene sets, along with focused enrichment in immune-inhibitory signaling pathways.

Gene Ontology enrichment analysis was conducted to examine biological functions associated with the targets of the 55 differentially expressed serum miRNAs. Enriched cellular component terms were primarily related to the cytoplasm/cytosol, plasma membrane, endoplasmic reticulum, Golgi apparatus, and extracellular space (Figure 3A). These results suggest that radon-associated miRNA changes are linked to processes involving cellular membranes, vesicle trafficking, and secretion.

For molecular functions, enriched terms included metal-ion binding, ATP binding, and acetylation-related activities, consistent with pathways involved in ion handling, cytoskeletal dynamics, and intracellular trafficking. In the biological process category, immune-modulatory pathways were prominent, including negative regulation of T-cell costimulation, IL-10 signaling, and Fc-receptor-mediated inhibitory signaling. Additional enrichment in prostaglandin metabolism and membrane depolarization suggests potential shifts in immune tone and ion-channel/electrophysiologic signaling in the high-radon group. The associated count plot (Figure 3B) shows that compartment-related terms reflect large gene sets (e.g., cytosol, cytoplasm, plasma membrane), whereas immune-inhibitory pathways reach significance with smaller but focused gene sets, consistent with targeted immune regulation rather than broad housekeeping effects.

### 3.5. KEGG Enrichment of Targets of Radon-Responsive miRNAs

**Figure 4:**
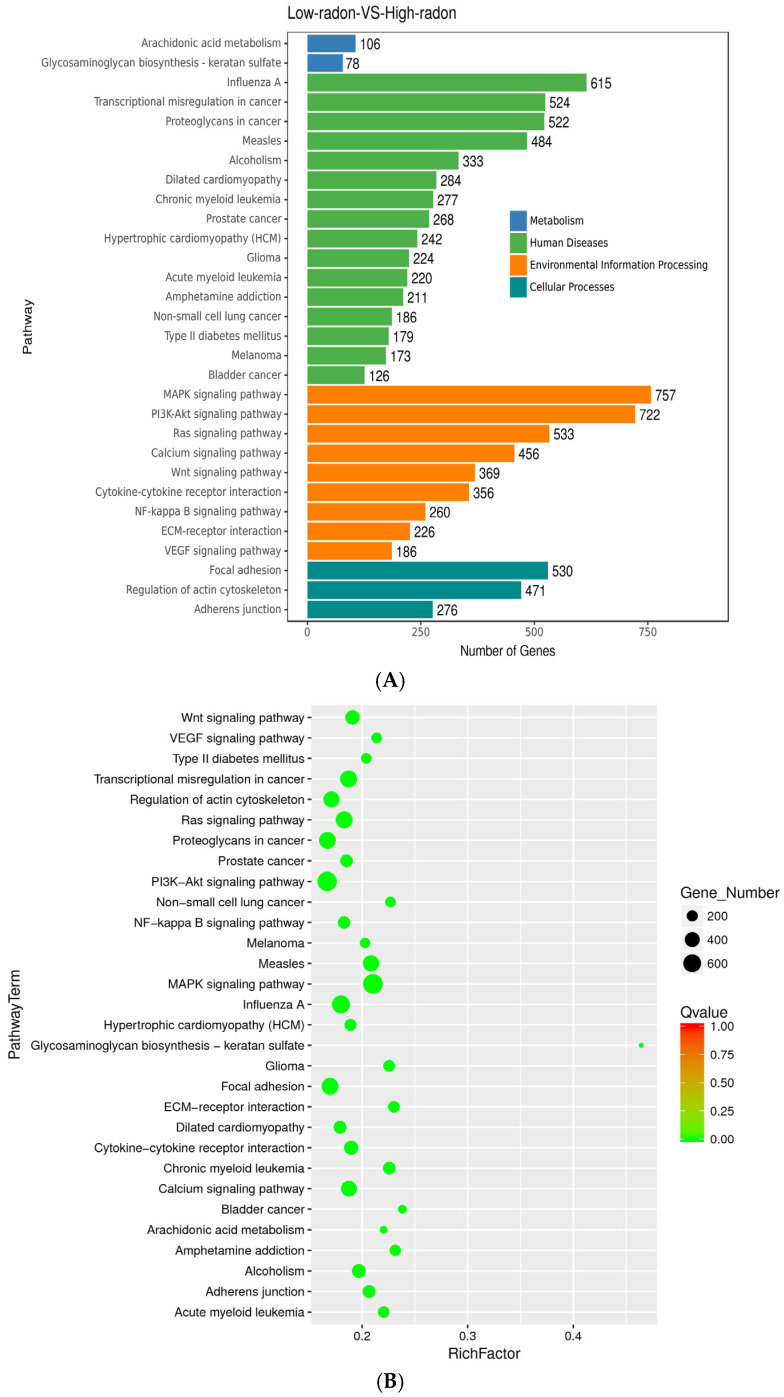
KEGG pathway enrichment of targets of radon-responsive miRNAs. (**A**) Bubble plot of the top enriched KEGG pathways for high vs low radon (targets of serum DE miRNAs). The x-axis shows RichFactor (hits ÷ pathway size); bubble area encodes the number of target genes; color indicates the FDR-adjusted q value. (**B**) Bar plot showing hit counts for the same pathways, grouped by KEGG parent category (Metabolism, Human Diseases, Environmental Information Processing, Cellular Processes). Notable signals include MAPK and PI3K–Akt (with Ras/Wnt/VEGF), focal adhesion/ECM–receptor interaction/regulation of actin cytoskeleton, cytokine/JAK–STAT/NF-κB pathways, and arachidonic acid metabolism.

KEGG (Kyoto Encyclopedia of Genes and Genomes) pathway enrichment of predicted mRNA targets highlighted a coherent set of signaling and cell structure/adhesion pathways. In the bubble plot (Figure 4A; ranked by RichFactor, bubble size = gene count, color = *q*-value), the leading pathways were MAPK and PI3K–Akt, with additional enrichment in Ras, Wnt, and VEGF signaling. Structural and adhesion modules were also prominent, including focal adhesion, ECM–receptor interaction, regulation of actin cytoskeleton, adherens junction, and calcium signaling, indicating coordinated regulation of growth factor signaling, adhesion, and cytoskeletal remodeling.

Immune and inflammation pathways were also enriched, including cytokine signaling and infection-related signatures (e.g., influenza A, measles), along with JAK–STAT and NF-κB signaling axes, alongside arachidonic-acid metabolism and proteoglycan/glycosaminoglycan pathways. These results align with GO findings, pointing to membrane/secretory processes and immune dampening in the high-radon group. The bar plot (Figure 4B) shows the highest hit counts in MAPK and PI3K–Akt, supporting an exposure-linked program that targets growth factor/adhesion and immune-modulatory networks. (Pathways with q < 0.05 were considered significant.)

### 3.6. Global Metabolomic Profiling of High- and Low-Radon Groups

**Figure 5:**
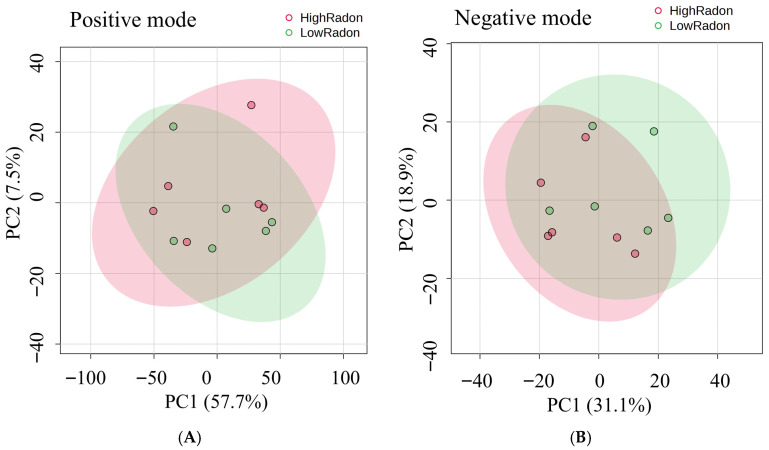
Principal component analysis (PCA) of serum metabolomic profiles by radon exposure. (**A**) Positive ion mode; (**B**) negative ion mode. Each point is one participant (pink = HighRadon, green = LowRadon); shaded ellipses indicate the 95% confidence region for each group. Axes show PC1 and PC2 with the percentage of variance explained (positive: PC1 = 57.7%, PC2 = 17.5%; negative: PC1 = 33.1%, PC2 = 18.9%). A modest separation with overlap is observed in both modes.

Serum metabolomic profiles from individuals residing in high- and low-radon exposure areas were characterized using LC–MS in both positive and negative ion modes. After rigorous QC filtering and normalization, the datasets were subjected to multivariate and univariate statistical analyses to explore group-wise differences. To assess overall differences in serum metabolomic patterns between high- and low-radon exposure groups, we first performed PCA (Figure 5). In the positive ion mode (Figure 5A), PC1 and PC2 explained 65.2% of the total variance (PC1 = 57.7%, PC2 = 7.5%), while in the negative ion mode (Figure 5B), they accounted for 52.0% (PC1 = 33.1%, PC2 = 18.9%). In both modes, samples from the two groups exhibited partial overlap but also showed a trend toward separation along PC1. This indicates that while radon exposure does not completely segregate individuals into distinct metabolic clusters, it induces measurable shifts in the global metabolomic landscape. Such patterns suggest that radon exposure may subtly but consistently alter systemic metabolic processes.

**Figure 6:**
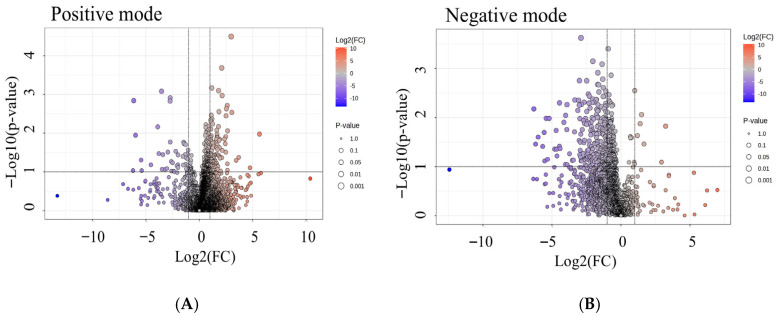
Volcano plots of differential metabolites by radon exposure. (**A**) Positive-ion mode; (**B**) negative-ion mode. Axes show log2(fold change) for HighRadon vs LowRadon (x) and −log10(*p* value) (y). Points are colored by log2FC (red = higher in HighRadon; blue = lower) and sized by significance (larger = smaller *p* value). Vertical gray lines mark the effect-size cutoffs (|log2FC| = 1); the horizontal line marks *p* = 0.05 (−log10p ≈ 1.3). Features beyond both cutoffs are considered significant.

To identify the specific metabolites driving these global differences, volcano plots were constructed for both ion modes (Figure 6). Each dot represents a metabolite feature, with the x-axis reflecting fold change (log2FC) and the y-axis showing statistical significance (−log10 *p*-value). Features to the right are more abundant in the high-radon group, while those to the left are enriched in the low-radon group. In both positive and negative modes (Figure 6A,B), numerous metabolites passed the thresholds for both significance and fold change, highlighting clear exposure-related differences. The distribution of significant features indicates that radon exposure perturbs multiple metabolic pathways, with several metabolites showing consistent enrichment in high-radon individuals. Together, these analyses reveal that radon exposure alters both the global structure of serum metabolic profiles and the abundance of specific metabolites, pointing to lipid metabolism, steroid biosynthesis, and nucleotide turnover as the pathways most affected.

### 3.7. Differential Metabolites Associated with Radon Exposure

Targeted analysis of significantly altered metabolites revealed distinct biochemical changes between low- and high-radon groups (Table 3 and Table 4). In the positive ion mode, steroid- and lipid-related metabolites were prominently affected. Polyunsaturated fatty acids, including docosahexaenoic acid (DHA), arachidonic acid, and γ-linolenic acid, also showed higher levels, reflecting the remodeling of membrane lipids and potential inflammatory activation. Elevated carnitine species (e.g., decanoyl-L-carnitine, lauroylcarnitine) indicated shifts in mitochondrial fatty acid β-oxidation, while changes in theobromine and phosphocholine suggested perturbations in methylation and phospholipid turnover.

In the negative ion mode, multiple features pointed to oxidative stress and nucleotide metabolism. Altered levels of eicosapentaenoic acid (EPA), 9-HEPE, 8-HETE, and 5,12-DiHETE highlight changes in oxylipin metabolism, consistent with enhanced lipid peroxidation under radon exposure. The nucleosides inosine and hypoxanthine were elevated, suggesting increased nucleotide turnover and DNA damage repair activity.

Together, these findings indicate that radon exposure is associated with a characteristic metabolic signature involving (i) remodeling of lipid and steroid metabolism, (ii) enhanced mitochondrial fatty acid utilization, (iii) activation of oxidative stress and DNA repair pathways, and (iv) altered amino acid metabolism. These alterations suggest systemic metabolic stress that may contribute to carcinogenesis and inflammatory processes in chronically exposed individuals. Consistent with the miRNA analysis, the higher-BMI participant in the high-radon group did not show metabolomic outlier behavior and did not appear to drive the observed radon-related metabolic differences.

## 4. Discussion

Small-area spatial mapping of lung cancer mortality in Chiang Mai (2008–2017) identified Hang Dong and the adjacent districts of Doi Lo and San Pa Tong as higher-risk areas, suggesting localized environmental contributors [5]. In this context, household radon measurements were collected in two Hang Dong communities (urban San Phak Wan and rural Nong Kaeo). Indoor radon concentrations centered around ~50–60 Bq/m^3^, and 6% of households (3/48) recorded ≥100 Bq/m^3^ (Figure 1). These findings are consistent with previous studies in Northern Thailand reporting annual means of 40–55 Bq/m^3^ and wider ranges of 17–118 to 26–322 Bq/m^3^ within an overall regional span of 11–405 Bq/m^3^ [4,15,16,17,18,19,20]. Concentrations in our study were generally below the 148 Bq/m^3^ action level, but slightly above the global averages cited in the Thai literature. Seasonal variation during the burning period and factors such as local geology and home ventilation likely contribute to this variability between households. Based on evidence of excess lung cancer risk at residential levels, exposure groups were defined using the WHO 100 Bq/m^3^ reference level, with households ≥100 Bq/m^3^ classified as high exposure and those <50 Bq/m^3^ as low exposure.

Indoor radon exposure was associated with coordinated changes in circulating miRNAs and serum metabolites, indicating a systemic molecular response. The radon-related miRNA signature was enriched in pathways involving PI3K/Akt, MAPK, Ras, VEGF, cell adhesion, cytoskeleton organization, and immune regulation. Corresponding metabolomic shifts were seen in lipid and steroid metabolism, β-oxidation, redox balance, and nucleotide turnover. Together, these patterns are consistent with known effects of α-particle radiation on airway epithelial cells and suggest a link between residential radon exposure and pathways relevant to early carcinogenic processes.

Radon progeny deposit in the airway epithelium and deliver high-LET α-particle radiation, producing DNA double-strand breaks, oxidative stress, and genomic instability [21]. This activates ATM-mediated DNA-damage responses, autophagy, and survival signaling [22]. Chronic radon exposure in bronchial cells has been shown to activate PI3K/Akt signaling [23], which interacts with Ras–MAPK pathways [23] and promotes VEGF expression and extracellular matrix remodeling [24,25,26]. The observed enrichment of PI3K/Akt, MAPK, Ras, VEGF, and cytoskeleton-related pathways in circulating miRNAs aligns with this mechanistic sequence. Evidence for Wnt signaling activation in radon-exposed lung models is limited, although it has been discussed in broader radiation-response literature [27].

Serum small RNA sequencing identified a distinct miRNA signature associated with higher household radon. The most pronounced changes were increased levels of miR-200b-3p, miR-200c-3p, and miR-194-5p, and decreased levels of miR-3913-5p, miR-584-5p, miR-30a-3p, miR-22-3p, and miR-125a-5p. These expression patterns are consistent with biological responses to airway stress and early carcinogenic processes. The miR-200 family (miR-200b/c) maintains epithelial identity by inhibiting ZEB1/ZEB2, key drivers of epithelial-to-mesenchymal transition (EMT)) [28,29]. Higher circulating levels, therefore, suggest a compensatory, anti-EMT response to radon-related epithelial injury rather than a tumor-promoting pattern. miR-194-5p, which suppresses invasion and regulates HIF-1α, was also increased, supporting a protective or repair-oriented response [30,31,32,33].

In contrast, several tumor-suppressive miRNAs decreased, which could reduce inhibitory control of pathways linked to motility, stress tolerance, and immune surveillance. Lower miR-584-5p may promote cell movement and extracellular vesicle release through loss of YKT6 regulation [34]. Reduced miR-30a-3p and miR-22-3p align with enhanced survival and EMT-related signaling; miR-22-3p also modulates MET/STAT3 and RAC1 pathways [35]. Decreased miR-125a-5p, known to influence immune-checkpoint signaling via IGSF11/VSIG3 [36], may reflect reduced baseline anti-tumor immune tone. miR-3913-5p, often elevated in tumor exosomes and therapy resistance, showed lower levels here, consistent with early exposure biology rather than malignant behavior [37]. Notably, the downregulation of miR-22-3p may be mechanistically linked to the observed elevation in oxylipins, as miR-22 is known to regulate PI3K/Akt and the oxidative stress pathways involved in lipid remodeling [38]. This miRNA pattern is consistent with experimental studies showing that repeated radon exposure in bronchial cells and mouse models activates the PI3K–AKT–mTOR pathway and induces EMT-like changes, mitochondrial stress, and p53-dependent responses. These pathways also interact with MAPK and angiogenic signaling, which is reflected in the pathway enrichment results observed here [31].

Serum metabolomics in the same participants showed consistent changes in lipid, energy, and nucleotide pathways. Higher levels of phosphocholine (membrane synthesis), acyl-carnitines (β-oxidation), polyunsaturated fatty acids and oxylipins (AA, DHA, 9-HEPE, 8-HETE, 5,12-DiHETE), and purine breakdown products (inosine, hypoxanthine) were observed. These patterns are compatible with oxidative stress, DNA-damage responses, PI3K/Akt–VEGF signaling, mitochondrial fatty-acid metabolism, and enhanced membrane turnover, processes that can accompany EMT and cytoskeletal remodeling [22,25,26]. An independent study in a high natural background area of Chiang Mai also reported altered sphingolipid and glycerophospholipid metabolism, including reduced D-sphingosine, in high-radon residents and lung cancer cases [39]. Decreased sphingosine aligns with radon-related oxidative stress and growth factor signaling, which can promote sphingolipid remodeling and conversion of sphingosine to S1P, shifting lipid balance toward pro-survival and angiogenic pathways. The observed increases in phosphocholine and oxylipins further support membrane turnover and inflammatory signaling. Together, these findings point to radon-associated membrane lipid remodeling as a shared biological response.

Structural changes that support cell movement and invasion also appear relevant. Experimental studies show that repeated radon exposure can induce EMT-like transitions, increased cell migration, and mitochondrial dysfunction in bronchial epithelial cells [30]. Radon-related radiation stress has also been shown to alter RAD9 signaling and reduce the expression of cytoskeletal-binding genes, indicating reorganization of the actin cytoskeleton [40]. These findings align with our pathway enrichment results involving focal adhesion and cytoskeleton regulation, and with the metabolomic increase in phosphocholine, consistent with enhanced membrane turnover during cell motility and growth.

The immune component of radon injury is supported by both these data and prior studies. Proteomic analyses of intermittently radon-exposed A549 cells show changes in stress-, mitochondrial-, and immune-related proteins [41]. In vivo and ex vivo studies demonstrate that radon-induced CCL5 (RANTES) recruits dendritic cells and activates effector T-helper cells after exposure, providing a mechanism for chronic inflammatory tone [42]. These observations align with the present miRNA enrichment in cytokine/JAK–STAT/NF-κB pathways and the metabolomic shift toward pro-inflammatory eicosanoids with partially pro-resolving mediators, consistent with PLA_2_/COX/LOX regulation downstream of PI3K/Akt and VEGF.

Although measured in serum, the molecular patterns observed here are consistent with events occurring in the airway epithelium. Radon-induced DNA damage response (DDR) and ATM activation in airway cells can alter miRNA expression, and extracellular vesicles and lipid mediators released from injured epithelium can enter circulation as exposure-linked signals [22,23,24]. In this context, elevated AA-derived oxylipins reflect lipid-mediator class switching and oxidative stress, while increased decanoyl- and lauroyl-carnitine suggests a shift toward β-oxidation as an adaptive response to reactive oxygen species [22]. Higher phosphocholine indicates enhanced membrane synthesis and turnover, consistent with EMT-related and cytoskeletal remodeling processes [30,40]. Accumulation of inosine and hypoxanthine reflects purine catabolism and DNA-repair activity following α-particle damage [22]. Together, these serum findings reflect biological responses downstream of airway injury, including oxidative stress, metabolic reprogramming, angiogenic and matrix changes, immune modulation, and genomic instability.

Taken together with the mechanistic evidence detailed above, the miRNA and metabolite findings showed clear points of convergence. Although this pilot study was not powered for formal multi-omics modeling, several radon-responsive miRNAs demonstrated pathway-level concordance with the metabolic alterations observed in serum. Upregulated miRNAs associated with oxidative stress, epithelial injury, and inflammatory activation (miR-200b-3p, miR-200c-3p, miR-194-5p) aligned with increases in arachidonic acid-derived oxylipins (HETE, HODE, 5,12-DiHETE), reflecting lipid peroxidation and membrane stress. Conversely, down-regulated miRNAs involved in metabolic stress regulation (miR-30a-3p, miR-22-3p, miR-125a-5p) coincided with elevated acyl-carnitines indicative of β-oxidation, and with higher inosine and hypoxanthine, consistent with purine turnover and DNA-damage responses. Together, these concordant miRNA–metabolite patterns support a radon-associated biology involving oxidative stress, membrane remodeling, and DDR/ATM-linked signaling.

**Figure 7:**
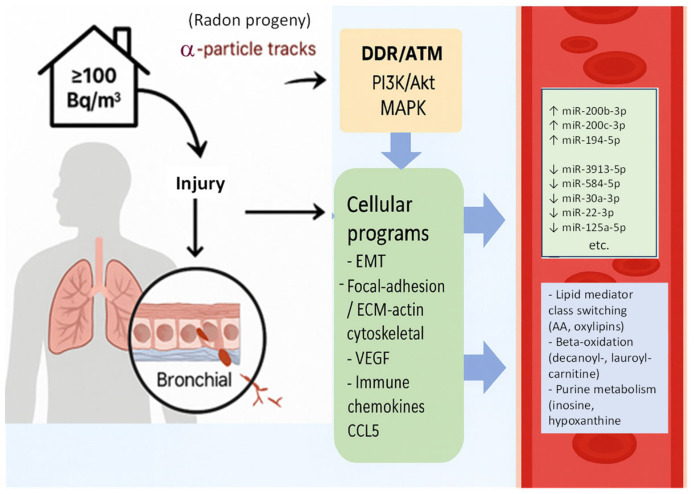
Proposed cascade of events linking residential radon exposure to circulating miRNA and metabolic signatures.

Figure 7 summarizes the proposed model in which household radon progeny deliver α-particle tracks to airway epithelium, activating DDR/ATM and PI3K/Akt–MAPK signaling, leading to adhesion and EMT remodeling, VEGF-driven angiogenesis, and immune activation (e.g., CCL5). The figure integrates the observed serum markers, including increased miR-200b-3p, miR-200c-3p, and miR-194-5p and decreased miR-3913-5p, miR-584-5p, miR-30a-3p, miR-22-3p, and miR-125a-5p, alongside metabolic changes such as oxylipin elevation, β-oxidation, phosphocholine upregulation, and purine turnover.

This study involved a small number of participants (12 collected; 10 included after exclusions), which limits its statistical power and generalizability. The cross-sectional design does not establish causality, and unmeasured factors (e.g., diet or other environmental exposures) may contribute to the results. Accordingly, these findings should be interpreted as exploratory and hypothesis-generating. Future studies should involve larger cohorts and incorporate single-cell RNA-seq and imaging mass spectrometry to map circulating signatures to specific cell types and tissue compartments, together with perturbation experiments in human airway models to test ATM→PI3K/Akt–mTOR and EMT/adhesion mechanisms under environmentally relevant radon levels.

Household radon (≥100 Bq/m^3^) yields α-emitting progeny that deposits in the bronchial epithelium, causing DNA double-strand breaks and oxidative stress. We posit activation of DDR/ATM converging on PI3K/Akt and MAPK hubs, driving EMT and adhesion/cytoskeletal remodeling, VEGF-linked angiogenesis, and immune-chemokine signaling (e.g., CCL5). In high-radon serum, miR-200b-3p, miR-200c-3p, and miR-194-5p were increased, whereas miR-3913-5p, miR-584-5p, miR-30a-3p, miR-22-3p, and miR-125a-5p were decreased. Metabolite changes included lipid-mediator class switching (AA-derived oxylipins), greater β-oxidation (acyl-carnitines such as decanoyl- and lauroyl-carnitine), and enhanced purine catabolism (inosine, hypoxanthine). Arrows denote hypothesized relationships; schematic not to scale.

## 5. Conclusions

In Chiang Mai, where lung cancer mortality risk is elevated, and residential radon typically ranges ~50–60 Bq/m^3^, higher household radon was associated with convergent serum miRNA and metabolomic patterns. Interpreted with prior evidence of bronchial epithelial injury from radon progeny, these signals are consistent with DDR/ATM activation, downstream PI3K/Akt–MAPK pathways, and resulting EMT/adhesion changes, VEGF-linked angiogenesis, and immune modulation—a biologically plausible bridge from exposure to early carcinogenic processes. From a public-health perspective, the findings support home testing and remediation at ≥100 Bq/m^3^, alongside continued tobacco-control efforts. Given Chiang Mai’s recurrent PM 2.5 episodes during the burning season [43,44], co-exposure may amplify oxidative stress and inflammatory lipid signaling; future work should use joint radon–PM study designs with high-resolution air-quality data. Circulating miRNAs and metabolites emerge as promising indicators of radon-related biological response. Future studies should validate in larger, longitudinal cohorts and incorporate extracellular vesicle analyses to define circulating molecular cargo more precisely.

## Figures and Tables

**Table 1:** Distribution of indoor radon concentrations by study area.

Area	N	Mean ± SD	Min–Max	No. of House Classified According to Radon Level (Bq/m^3^) WHO Reference = 100 Bq/m^3^	
				<100	≥100
San Phak Wan	25	64.5 ± 18.42	34–125	24	1
Nong Kaeo	23	60.0 ± 18.26	40–117	21	2
Both area	48	61.8 ± 18.36	34–125	45	3

Values are reported as mean ± SD and range (min–max) in Bq/m^3^. Households are classified by the WHO indoor radon reference level (100 Bq/m^3^) as follows: below reference (<100 Bq/m^3^) and at/above reference (≥100 Bq/m^3^).

**Table 2:** Significantly (padj < 0.05) differentially expressed serum miRNAs between low- and high-radon exposure groups.

Gene_id	log2 Fold Change	padj
miRNA significantly up-regulated		
NovelmiRNA-616 (ugugguucuagagagcucuuuugu)	27.7	9.34 × 10^−17^
2. NovelmiRNA-203 (gggguggggucugaggauuuguga)	26.7	6.88 × 10^−16^
3. hsa-miR-200b-3p	25.6	1.02 × 10^−14^
4. hsa-miR-200c-3p	24.5	1.10 × 10^−13^
5. hsa-miR-194-5p	23.3	1.88 × 10^−12^
6. hsa-miR-224-5p	8.7	0.034468
7. hsa-miR-93-5p	8.2	0.034885
8. hsa-miR-125b-5p	7.3	0.057078
9. hsa-miR-128-3p	7.0	0.052435
10. hsa-let-7g-5p	5.5	0.060395
11. hsa-let-7c-5p	4.3	0.045629
12. hsa-miR-21-5p	3.1	0.004513
miRNA significantly down-regulated		
hsa-miR-3913-5p	−24.4	5.52 × 10^−14^
2. hsa-miR-584-5p	−11.3	0.001881
3. hsa-miR-30a-3p	−10.9	0.000125
4. hsa-miR-22-3p	−10.3	0.00707
5. hsa-miR-125a-5p	−10.1	0.00192
6. hsa-miR-589-5p	−9.5	0.018719
7. hsa-miR-126-3p	−9.3	0.02387
8. hsa-miR-215-5p	−9.2	0.023942
9. hsa-miR-6852-5p	−9.1	0.023685
10. hsa-miR-500a-3p	−8.8	0.027473
11. hsa-miR-2110	−8.7	0.027473
12. hsa-miR-582-3p	−8.4	0.034885
13. hsa-miR-193a-5p	−8.3	0.034468
14. hsa-miR-382-5p	−8.2	0.034885
15. hsa-miR-150-3p	−8.2	0.034468
16. hsa-miR-671-3p	−8.2	0.034468
17. hsa-miR-139-3p	−8.1	0.034468
18. hsa-miR-152-3p	−8.1	0.042125
19. hsa-miR-11400	−8.0	0.035285
20. hsa-miR-654-5p	−7.9	0.039445
21. hsa-miR-129-5p	−7.6	0.042125
22. hsa-miR-148a-3p	−3.4	0.000174

For each miRNA, the gene identifier, log2 fold change (high vs. low radon), and adjusted *p*-value (padj < 0.05) are shown. Positive log2 fold change values indicate up-regulation in the high-radon group, while negative values indicate down-regulation. Novel miRNAs identified in this study are denoted as “NovelmiRNA”.

**Table 3:** Differentially abundant metabolites between low- and high-radon exposure groups (positive ion mode) *.

Met ID	RT (min)	*m*/*z*	Met Name	*p*-Value	*q*-Value (FDR-BH)	Low Radon (avg)	High Radon (avg)
2946	12.231	334.2	resorcinol (pregnenolone)	0.0001	0.011	7444	8000
3117	10.175	343.2	(Resolvin D5	0.0006	0.011	340,426	375,290
7656	9.5	584.3	Shearinine F (PC20:4/2:0)	0.0008	0.0012	41,331	48,311
2688	8.1	316.2	Decanoyl-L-Carnitine	0.0026	0.0036	874,330	1,922,109
1292	4.0	211.1	Cyclo (Leu-Pro)	0.0103	0.0032	46,651	205,776
2867	12.9	329.2	Docosahexaenoic acid	0.006	0.0078	37,253	33,172
2130	10.7	279.2	Gamma-Linolenic acid	0.0254	0.0206	10,057	32,302
2833	13.2	327.2	Arachidonic acid	0.0266	0.0144	130,013	174,315
970	2.1	181.0	Theobromine	0.0337	0.0435	122,185	388,356
1011	12.7	184.0	Phosphocholine	0.0367	0.0399	640,531	988,649
3147	9.5	344.2	Lauroyl carnitine	0.0491	0.0436	274,556	420,792

* Metabolites were identified by retention time (RT, min) and mass-to-charge ratio (*m*/*z*). Values represent average peak intensities in each group. Group differences were assessed using the Mann–Whitney U test; shown are nominal *p*-values and Benjamini–Hochberg FDR-adjusted q-values for each metabolite.

**Table 4:** Differentially abundant metabolites between low- and high-radon exposure groups (negative ion mode) *.

Met ID	RT (min)	*m*/*z*	Met Name	*p*-Value	q-Value (FDR-BH)	Low Radon (avg)	High Radon (avg)
2130	13.0	301.2	Eicosapentaenoic acid	0.0062	0.0103	247,489	322,314
2335	13.6	317.2	9-HEPE	0.0043	0.0103	48,350	79,112
2356	13.0	319.2	8-HETE	0.005	0.0103	1,853,013	2,266,134
1746	1.3	267.0	Inosine	0.0041	0.0103	300,249	683,873
2336	12.2	317.2	5-HEPE	0.0056	0.0103	125,978	149,332
2552	10.5	335.2	5,12-DiHETE	0.0032	0.0103	3,379,115	4,049,347
2059	12.2	295.2	9-HODE	0.0115	0.0164	1,091,002	1,395,424
4088	11.7	455.3	Ursolic acid	0.0375	0.0422	29,786	39,853
442	0.89	135.0	Hypoxanthine	0.038	0.0422	76,322	153,048
1872	12.2	277.2	γ-Linolenic acid	0.0517	0.0517	53,311	76,549

* Metabolites were identified by retention time (RT, min) and mass-to-charge ratio (*m*/*z*). Values represent average peak intensities in each group. Group differences were assessed using the Mann–Whitney U test; shown are nominal *p*-values and Benjamini–Hochberg FDR-adjusted q-values for each metabolite.

## Data Availability

Data are available from the corresponding author on reasonable request.

## Supplementary Materials

The following supporting information can be downloaded at: https://www.mdpi.com/article/10.3390/toxics13121021/s1, Figure S1. Validation of small RNA seq results by realtime RT-PCR.

## Abbreviations

The following abbreviations are used in this manuscript:

AA	arachidonic acid
AC	acyl-carnitine
AKT	protein kinase B
ATM	ataxia telangiectasia mutated
Bq/m^3^	becquerels per cubic meter
CCL5 (RANTES)	C-C motif chemokine ligand 5
COX	cyclooxygenase
DDR	DNA-damage response
DESeq2	Differential Expression analysis for sequence count data
DHA	docosahexaenoic acid
DiHETE	dihydroxyeicosatetraenoic acid
DNA	deoxyribonucleic acid
DNMT3A	DNA methyltransferase 3A
EGFR-TKI	epidermal growth factor receptor tyrosine kinase inhibitor
EMT	epithelial–mesenchymal transition
EPA	eicosapentaenoic acid
ESI	electrospray ionization
EV	extracellular vesicle
FDR	false discovery rate
GO	Gene Ontology
HEPE	hydroxyeicosapentaenoic acid
HETE	hydroxyeicosatetraenoic acid
HIF-1α	hypoxia-inducible factor-1 alpha
HODE	9-hydroxyoctadecadienoic acid
IGSF11	immunoglobulin superfamily member 11
JAK–STAT	Janus kinase–signal transducer and activator of transcription
KEGG	Kyoto Encyclopedia of Genes and Genomes
LC–MS	liquid chromatography–mass spectrometry
LET	linear energy transfer
log2FC	log2 fold change
LOX	lipoxygenase
MAPK	mitogen-activated protein kinase
MS1	precursor (MS^1^) scan
MS2	fragment (MS^2^) scan
mSv·y^−1^	millisieverts per year
MMP	matrix metalloproteinase
mTOR	mechanistic/mammalian target of rapamycin
NF-κB	nuclear factor kappa-light-chain-enhancer of activated B cells
NGS	next-generation sequencing
nt	nucleotides
padj	FDR-adjusted *p*-value
PCA	principal component analysis
PC	phosphatidylcholine
PI3K	phosphoinositide 3-kinase
PLA_2_	phospholipase A_2_
PM_2_._5_	fine particulate matter ≤2.5 μm
PUFA	polyunsaturated fatty acid
q value	FDR-adjusted *p*-value
RT-qPCR	reverse transcription quantitative PCR
RAC1	Ras-related C3 botulinum toxin substrate 1
RAD9	cell-cycle checkpoint protein RAD9A
ROS	reactive oxygen species
RT (chromatography)	retention time
S1P	sphingosine-1-phosphate
VEGF	vascular endothelial growth factor
VSIG3	V-set and immunoglobulin domain-containing 3
Wnt	wingless-related integration site pathway
YKT6	vesicle SNARE protein YKT6
ZEB1	zinc finger E-box-binding homeobox 1
ZEB2	zinc finger E-box-binding homeobox 2
