# Supplementary material for: Serum miRNA and Metabolomic Signatures of Residential Radon Exposure in Chiang Mai, Thailand
Source: Toxics. 2025 Nov 26;13(12):1021. doi: 10.3390/toxics13121021 (PMC12737089; doi:10.3390/toxics13121021)
Supplement: Supplementary file 1 [file toxics-13-01021-s001.zip › toxics-3928625-supplementary.pdf]

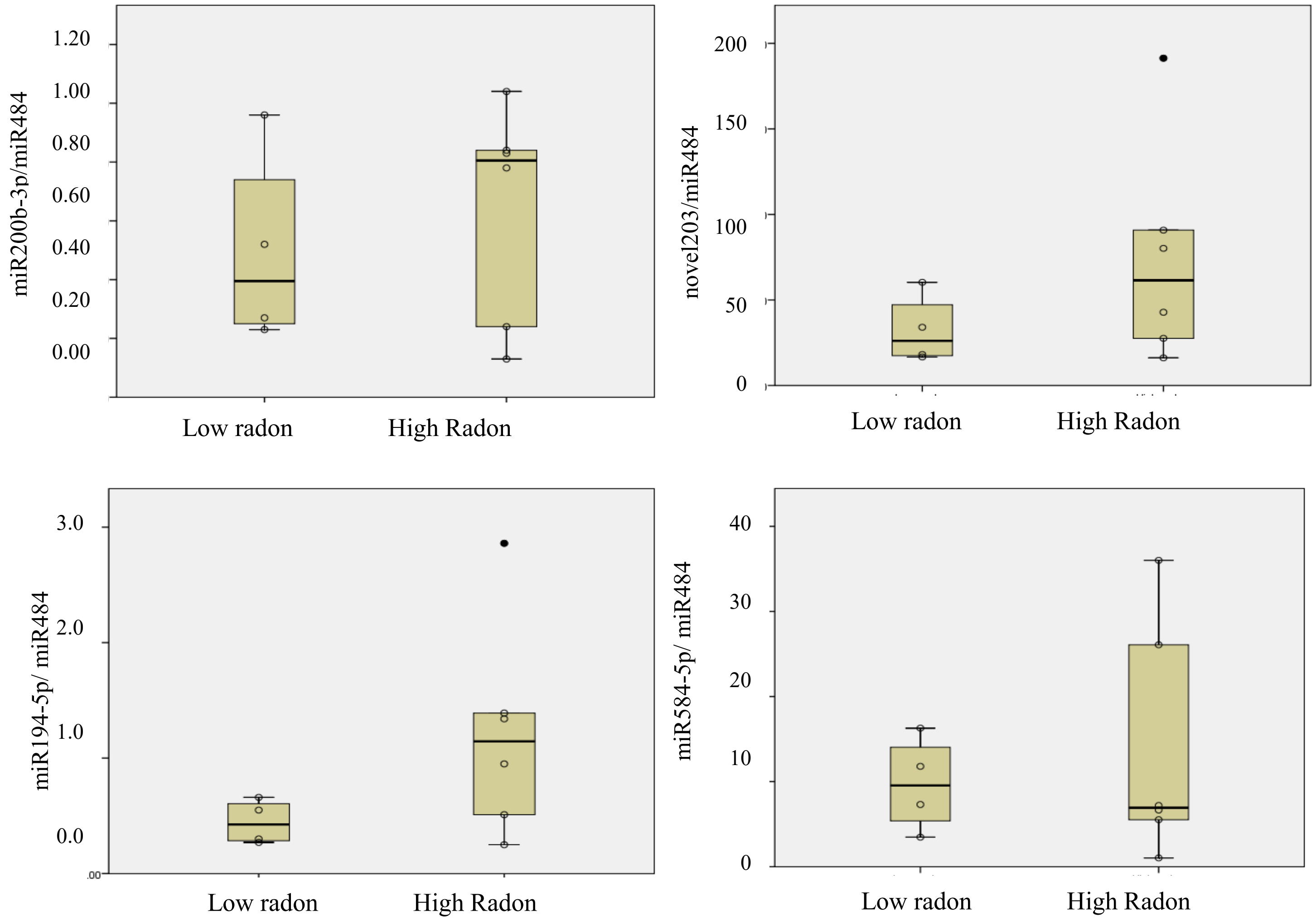

Supplementary Figure S1. Validation of small RNA seq results by realtime RT-PCR

Serum miRNAs from the same 10 non-smoking volunteers profiled by small RNA-seq were quantified by RT-qPCR and stratified by household radon level using the WHO reference threshold (Low radon: <100 Bq/m³, n = 4; High radon: ≥100 Bq/m³, n = 6). Expression of selected miRNAs—miR-200b-3p, Novel-miRNA-203, and miR-194-5p (upregulated in sequencing) and miR-584-5p (downregulated in sequencing)—was normalized to miR-484 and plotted as relative expression ($2^{-\Delta Ct}$, linear scale). Boxplots display the interquartile range (IQR), median (horizontal line), 1.5×IQR whiskers, and individual data points (solid circles denote values outside the whiskers). Although not statistically significant, consistent trends with the RNA-seq discovery analysis are observed, supporting technical validation of key miRNA candidates.
